# *CLN8* Mutations Presenting with a Phenotypic Continuum of Neuronal Ceroid Lipofuscinosis—Literature Review and Case Report

**DOI:** 10.3390/genes12070956

**Published:** 2021-06-23

**Authors:** Magdalena Badura-Stronka, Anna Winczewska-Wiktor, Anna Pietrzak, Adam Sebastian Hirschfeld, Tomasz Zemojtel, Katarzyna Wołyńska, Katarzyna Bednarek-Rajewska, Monika Seget-Dubaniewicz, Agnieszka Matheisel, Anna Latos-Bielenska, Barbara Steinborn

**Affiliations:** 1 Chair and Department of Medical Genetics, Poznan University of Medical Sciences, 60-352 Poznan, Poland; contact@br-ai-n.org (A.S.H.); kat.wolynska@gmail.com (K.W.); alatos@ump.edu.pl (A.L.-B.); 2 Chair and Department of Developmental Neurology, Poznan University of Medical Sciences, 60-355 Poznan, Poland; awwiktor@ump.edu.pl (A.W.-W.); bstein@ump.edu.pl (B.S.); 3 Department of Neurology, 10th Military Research Hospital and Polyclinic, 85-681 Bydgoszcz, Poland; apiet@o2.pl; 4 BIH Genomics Core Unit, Campus Mitte, Charite University Medicine, 13353 Berlin, Germany; tomasz.zemojtel@charite.de; 5 Department of Clinical Pathology, Poznan University of Medical Sciences, 60-355 Poznan, Poland; szlapus@hotmail.com (K.B.-R.); mseget@ump.edu.pl (M.S.-D.); 6 Department of Developmental Neurology, Gdansk Medical University, 80-307 Gdansk, Poland; ajuzwa@gumed.edu.pl

**Keywords:** *CLN8*, LINCL, lipofuscinosis, mutation, neuronal, ceroid

## Abstract

CLN8 is a ubiquitously expressed membrane-spanning protein that localizes primarily in the ER, with partial localization in the ER-Golgi intermediate compartment. Mutations in *CLN8* cause late-infantile neuronal ceroid lipofuscinosis (LINCL). We describe a female pediatric patient with LINCL. She exhibited a typical phenotype associated with LINCL, except she did not present spontaneous myoclonus, her symptoms occurrence was slower and developed focal sensory visual seizures. In addition, whole-exome sequencing identified a novel homozygous variant in *CLN8*, c.531G>T, resulting in p.Trp177Cys. Ultrastructural examination featured abundant lipofuscin deposits within mucosal cells, macrophages, and monocytes. We report a novel *CLN8* mutation as a cause for NCL8 in a girl with developmental delay and epilepsy, cerebellar syndrome, visual loss, and progressive cognitive and motor regression. This case, together with an analysis of the available literature, emphasizes the existence of a continuous spectrum of *CLN8*-associated phenotypes rather than a sharp distinction between them.

## 1. Introduction

Neuronal ceroid lipofuscinoses (NCLs) are a group of hereditary lysosomal storage disorders causing a progressive neurodegenerative disease that manifests with motor and cognitive deterioration, seizures, vision loss and ultimately shortened life expectancy [1]. They are characterized by intracellular accumulation of autofluorescent lipopigment reminiscent of the well known lipofuscin associated with normal aging and various pathological processes [2]. Relatively rare as individual disease entities, taken together, NCLs comprise the most common cause for progressive neurodegenerative disease in children. In the past, they were classified according to the age of onset as infantile, late-infantile, juvenile and adult NCL, also known under eponyms denoting the authors of the original reports Table 1. The NCL term was suggested in 1969 by Zeman and Dyken [3].

Since then, several additional forms have been described, including variants of the classic phenotypes, a congenital form and a progressive epilepsy syndrome. The disease’s genetic background continues to unravel. Thus far, more than 430 variants in at least candidate 13 genes (*CLN1*-*CLN14*) are recognized, all autosomal recessive except for the autosomal dominant NCL4, *DNAJC5* mutations responsible for the adult onset Parry disease [14,15] NCLs share the common features of cognitive deterioration, movement disorders (including cerebellar ataxia, myoclonus and spasticity), seizures and, in most instances, vision loss due to retina and optic nerve involvement. The exact phenotype, however, depends on the causative gene, mutation and resulting pathology. There is significant phenotypic, genetic and allelic variability, but some genotype-phenotype correlates have been established [16]. A new unifying classification was proposed by Williams et al. [17]. The most prevalent forms are caused by mutations in *CLN2* and *CLN3*, usually presenting as classic late infantile and classic juvenile phenotypes, respectively. The late infantile NCL in its classic form is caused by *CLN2* mutations. It presents between 2 and 4 years of age with epileptic seizures, soon accompanied with cognitive regression, myoclonus and cerebellar ataxia; visual loss usually develops over the following 2 years [1]. However, the late infantile NCL is also the most heterogenous, with several variants and at least three other causative genes (*CLN5*, *CLN6* and *CLN8*) [16,18]. Among the less common NCL forms, there is much heterogeneity and many uncertainties. Not uncommonly, this sets the stage for erratic workup and diagnostic surprises. We report a novel *CLN8* mutation causing a variant late-infantile neuronal ceroid lipofuscinosis in the index patient.

## 2. Materials and Methods

The whole-exome sequencing was applied (SureSelect XT, Agilent, Santa Clara, CA, USA). The sequencing was done on the next-generation platform (HiSeq, Illumina, San Diego, CA, USA). The discovered variants have been analyzed with variant prioritization software Exomiser/PhenIX as Zemojtel et al. described [19]. The following HPO terms were used as input: seizures, abnormality of the hindbrain. >96% of the target region was covered with at least 20 reads. We limited our evaluation to the top 20 ranked genes as described by Zemojtel et al. [19].

## 3. Results

### 3.1. Case Presentation

**Figure 1:**
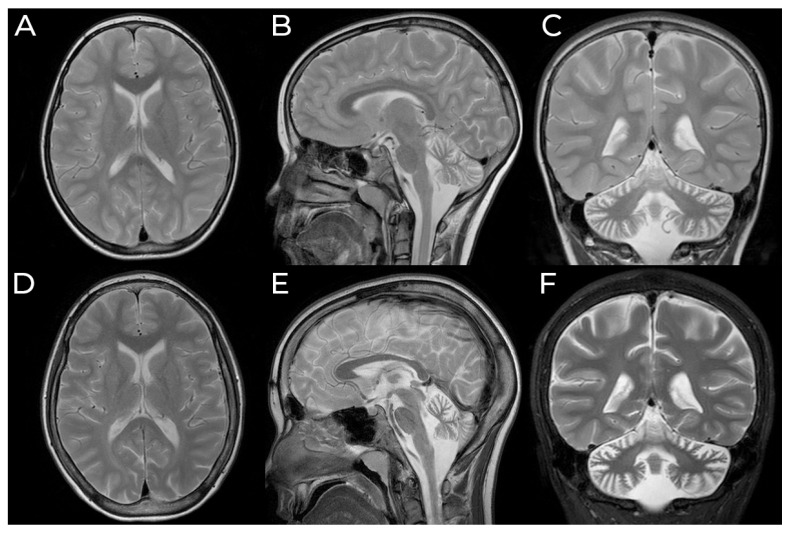
Brain MRI (T2-weighted, axial, sagittal, and coronal planes) of the patient with a mutation c.531C>T in the *CLN8* gene, at the age of 8 years (**A**–**C**) and the age of 12 years (**D**–**F**) showing bilateral periventricular white matter hyperintensities and progressive cerebellar atrophy.

A 13-year old girl was referred to our clinic due to progressive cerebellar ataxia, epilepsy, and bilateral optic nerve atrophy. Her non-consanguineous parents are in good health. Also, her 4-year older brother remains unaffected. The patient was born from uncomplicated pregnancy, with the Apgar score of 10/10. She required only 2 days of phototherapy for neonatal jaundice. Unfortunately, with time the patient started to manifest developmental delay. Although she could sit at the age of 6 months, she did not walk until 22 months. Moreover, until 3 years, her speech was limited to single words. As a calm child, she adapted well to pre-primary school, and with continuous therapy, her language production and comprehension were improving. At the age of 5.5, she experienced her first epileptic seizure being unresponsive and flaccid for 10–15 min. After a month, she had a nocturnal tonic-clonic seizure. An interictal EEG featured runs of temporal slow waves with no sharp transients. The diagnosis of focal epilepsy was made, and the valproate treatment was initiated. Initially, the seizures were rare, with a total of 3–4 over the next 2 years. At the age of 8, a worsening was induced by carbamazepine addition. An MRI obtained at that time showed bilateral periventricular white matter T2/FLAIR hyperintensities and cerebellar atrophy (Figure 1). The combined therapy with valproate, levetiracetam, and lacosamide prevented further seizures for almost 2 years. Cognitive developmental delay was apparent by that time. She presented abstract thinking and reasoning deficits, good long-term memory and social skills, and a slight dysarthric speech. At the age of 9.5, the deterioration of visual acuity was noted. An ophthalmic assessment disclosed bilateral optic nerve atrophy, a finding further confirmed by nerve thinning on MRI and marked conduction disturbance in visual evoked potentials study (VEP). Retinal fluorescein angiography revealed concurrent macular dystrophy. The brain MRI showed a progression of cerebellar atrophy (Figure 1). The neurological assessment noted an inappropriately cheerful attitude, mild hypotonia, and clumsiness of movement. Her cognitive performance deteriorated slowly to moderate impairment. By the age of 12, the patient presented with mood changes, occasional aggression, and particular hypersensitivity to loud sounds. Seizures became more frequent and occurred once every 3 weeks, as prolonged focal seizures with intense fear and elementary visual hallucinations (colorful dots and flashes). They could last up to an hour and secondarily generalize to a bilateral tonic-clonic seizure. On neurological examination, frank limb and truncal ataxia with pronounced dysarthria were present. Brain MRI and spectroscopy results were comparable to the previous ones. A trial of add-on lamotrigine was performed, resulting in increased seizure frequency and changed morphology. The seizures included now limbs myoclonias. Control EEG revealed a poorly organized, markedly slowed (4 Hz) background rhythm, with numerous high voltage slow waves. Notably, a generalized discharge of 2–3 Hz slow waves intermixed with sharply contoured transients was present.

### 3.2. Molecular and Histological Testing

**Figure 2:**
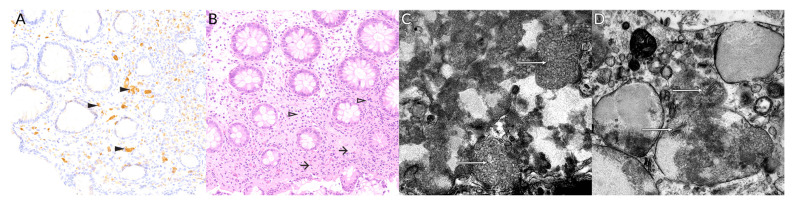
(**A**) Increased number of CD68 positive interstitial macrophages loaded with lipofuscin within colorectal mucosal biopsy (black arrowheads). Original magnification 200×. (**B**) Colorectal mucosa biopsy showing an increased number of interstitial macrophages (arrows) and a slightly increased amount of eosinophilic stroma of the lamina propria (hollow arrowheads). Original magnification 200×. Electron micrograph shows the biopsy of the rectal mucosa with the abnormal deposits in the epithelial cells and macrophages presenting as (**C**) the curvilinear profiles (white arrows), and (**D**) the fingerprint profiles (white arrows). Original magnification 105,000×.

For the suspected NCL2/Batten disease, the Sanger sequencing of *CLN2* gene was performed but did not reveal any changes. Exome sequencing detected a homozygous variant in *CLN8* gene, c.531G>T:p.(Trp177Cys), hg19 genomic position chr8:g.1719751G>T. Coverage at the position of this variant was >90 reads. PhenIX ranked the *CLN8* variant in the 1st place. This allele was neither previously reported in NCL nor featured in ExAC and 1000G databases. The mutation is located within a highly conserved sequence; this, together with the results of two proteomic analyses, suggests its pathogenicity. Both parents are carriers of the variant. To confirm the diagnosis of NCL, a rectal mucosal biopsy was obtained. The routine optic microscopic showed minor disturbance of mucosal architecture and unspecific, mild, chronic inflammation with an increased number of interstitial macrophages and a slightly increased amount of stroma within lamina propria.The routine optic microscopic study was unremarkable, showing minor disturbance of mucosal architecture and unspecific, mild, chronic inflammation. Ultrastructural examination featured abundant lipofuscin deposits within mucosal cells, macrophages, and monocytes, at varying stages of condensation, as well as extracellular storage material released from the most burdened, collapsed macrophages. Figure 2 shows some of the obtained results.

### 3.3. Follow-Up

The patient is currently 13 years old. Although the cerebellar syndrome continues to progress, she remains ambulatory. Her cognitive impairment was graded moderate in a recent neuropsychological assessment, with a major contribution of attention deficits. Visual acuity was estimated at 0.1 and 0.05 for the right and left eye, respectively. Neurological examination reveals wide-based gait, truncal and limb ataxia, hypotonia, and scanning speech, as well as knee and ankle contractures, anterocollis, and sluggish pupillary light reflex. Current EEG recording discloses interictal bilateral sharp and slow wave complexes, superimposed over a slow background with predominant 5–6 Hz theta and low amplitude beta rhythms. Considering the clinical features of epilepsy, cerebellar and optic nerve atrophy, macular dystrophy, developmental delay and progressive deterioration together with a *CLN8* mutation and relevant ultrastructural pathology, the diagnosis of neuronal ceroid lipofuscinosis type 8 was made.

## 4. Discussion

Mutations in *CLN8* are responsible for two main phenotypes. First one is the Northern Epilepsy (OMIM:610003), a distinctive progressive myoclonic epilepsy described in Finland [20,21]. The second variant is late-infantile NCL (OMIM:600143) reported primarily in Turkey [22]. Northern epilepsy syndrome (NE, also known as progressive epilepsy with mental retardation, EPMR), the first entity associated with *CLN8* in humans, belongs to a collection of autosomal recessive diseases over-represented among a remote population in northern Finland due to founder effect and relative isolation. This entity combines epilepsy, progressive mental deterioration, and cerebellar atrophy. In contrast to other NCLs, neither myoclonus nor retinal degeneration is a feature of EPMR. Together with longer life expectancy, this places EPMR between classic childhood NCLs with its early onset and Kufs disease with the absence of retinal involvement [23]. The initially reported 26 cases originated from a single pair of ancestors and carried a homozygous missense *CLN8* variant (C70G; Arg24Gly) [20]. However, a similar phenotype was recently identified in a large consanguineous Turkish family with their own novel *CLN8* mutation [24]. The disease manifests at the age of 5–10 years with generalized tonic-clonic seizures, with an initial frequency of one seizure every 1–2 months, aggravating at puberty and becoming less frequent but persistently present in adulthood. One-third of the patients experience complex focal seizures as well, but curiously, no myoclonus is reported. Approximately 2–5 years after the onset of seizures, a rapid cognitive decline is noted. Although it peaks at periods of high epileptic activity, the deterioration continues relentlessly even with good seizure control, leaving the patient moderately to profoundly impaired in adulthood. Neurological status is initially normal, but with time, cerebellar ataxia emerges, with clumsiness, balance disturbance, and dysarthric speech. While no ocular anomaly is apparent, a mild visual acuity decrease may be present. Predominately neuronal, autofluorescent deposits confirm the disease’s classification among NCLs [25]. It has recently been shown that this accumulation is not directly correlated with the degree of neuronal loss [26]. While the storage material is present in all neurons, its distribution is markedly uneven. Large amounts are found in layer III of isocortex and CA2, CA3, and CA4 hippocampal fields, with relative sparing of other isocortex layers and hippocampal CA1 as well as the cerebellar cortex, profoundly affected in other NCLs [25]. The other mentioned phenotype associated with *CLN8* mutation is a late infantile NCL variant (vLINCL) [27]. In contrast to both Finnish and Turkish EPMR patients, these patients have unequivocal retinal involvement. The onset is between 2 and 7 years of age, with rapid developmental regression, ataxia, myoclonus and epilepsy, and a prompt visual failure. Subsequent cases were reported in Turkey [18,22], Italy [28], Germany [29], Israel [30], Ireland [31], Pakistan [22], Japan [32], China [33], and Saudi Arabia [34], some showing a milder phenotype. A few highly atypical presentations reported thus far include a congenital NCL in an Argentinian female [35] and a seizure disorder with retinitis pigmentosa but normal cognition in Hispanic siblings [36]. Table 2 summarizes these reports.

**Figure 3:**
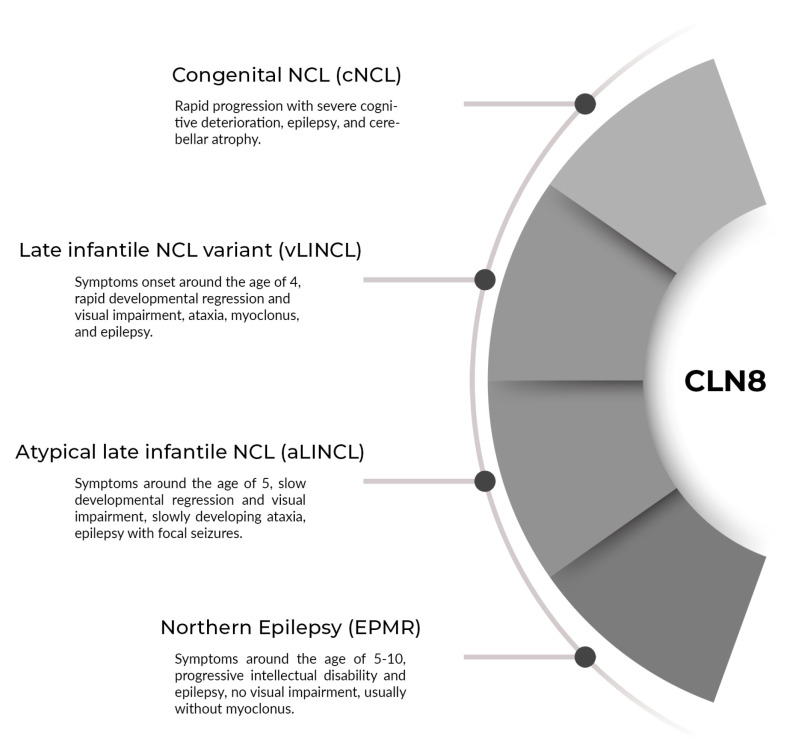
The proposed spectrum of NCL8 clinical phenotypes.

For vLINCL, the mean age at onset is 5 years (range 2–16 years, excluding the congenital case). EPMR begins later, in the seventh or ninth year of life, in the reported Finnish and Turkish populations, respectively. Our case had a typical time of onset at the age of 5 and a half. For most patients, vLINCL is rapidly progressive, leading to severe cognitive impairment and loss of mobility in as little as 2–4 years. On the other hand, the progression rate is much slower in EPMR, with the patients less demented and still walking in middle adulthood. However, as the *CLN8* gained recognition, several otherwise typical vLINCL families were reported with more indolent courses resembling EPMR. Thus, our case appears to belong to them as well. Overall, in NCLs, the onset of the disease is usually reported at the emergence of the more indicative symptoms of epilepsy, ataxia, visual impairment, or cognitive decline. They may occur either in the background of normal or delayed development. Our case belongs to the latter group. Over half of NCL8 vLINCL cases (15 out of 24 specified) displayed some delay of speech and gait; there is one report of severe congenital retardation [35]. On the contrary, normal development before seizures is typical in EPMR (25 out of 28 patients). With the exclusion of developmental delay, the first symptoms are epileptic seizures in most cases. Our patient experiences at least two types of seizures, generalized tonic-clonic (GTCS) and focal seizures with impaired awareness and occipital phenomenology. While all known NCL8 cases feature some generalized seizure patterns (GTCS, myoclonic, atonic, or absence), focal seizures were reported infrequently. They were present in 30% of original EPMR patients but apparently absent in the Turkish EPMR kindred. Among vLINCL, focal epileptiform discharges were noted in the Irish case only [31]. Unfortunately, no details of seizure phenomenology were given. Nevertheless, the unequivocally focal seizures with visual aura followed by awareness disturbance and occasional secondary generalization distinguish our patient from the NCL8 presentations reported thus far. It is also worth noting that no spontaneous myoclonus was present in our case. Myoclonias are a typical feature of NCLs. Curiously, they were absent in EPMR and, apparently, in the Irish vLINCL case - in both, focal seizures or EEG discharges were reported instead [31]. Our patient developed jerky involuntary movements, possibly myoclonias, on lamotrigine, a sodium channel blocker notorious for worsening myoclonus. In some progressive myoclonic epilepsies, myoclonic jerks may be elicited by specific stimuli and accompanied by a giant sensory potential of respective modality in neurophysiological studies [37]. Likewise, noise-evoked myoclonus was reported in the Pakistani boy by Reinhardt et al. [29]. Our patient is susceptible to loud sounds; however, she does not present myoclonus. Her brainstem auditory evoked potentials are normal as well. Visual loss due to retinal degeneration is a cardinal feature of childhood NCLs, including NCL8 vLINCL, where the acuity begins to deteriorate around age 6 (range: 2–13) and is usually rapid and severe. Associated retinal pathology varies and may involve both peripheral or macular retina. In many cases, optic nerve atrophy was noted and documented by pale optic discs on fundoscopy and abnormal visual evoked potentials (VEP). EPMR considered the milder end of the NCL8 disease spectrum, distinctively lacks apparent retinal involvement. However, minor visual disturbance without frank retinal pathology is present in occasional patients (7 out of 16) - most likely due to optical nerve atrophy, as VEP were abnormal in these cases. Our patient developed macular and optic dystrophy with significant visual impairment; however, this occurred relatively late compared to other vLINCL reports. Another component of the NCL8 disease spectrum is cerebellar syndrome with ataxia and dysarthria. It usually emerges close to the onset of seizures and worsens rapidly. Together with cognitive impairment, it is the major contributor to disability in vLINCL, rendering the patient chairbound and mute. Conversely, in EPMR cases and our patient, the cerebellar syndrome is present but progresses much more slowly. Our patient used to ride a bicycle until the age of 11, remains ambulatory and capable of comprehensible speech. Cognitive deterioration accompanies other symptoms of NCL in all but one reported family [36], but the rate and severity of impairment vary greatly. Overall, it appears to parallel the general course of the disease, with the more rapidly progressive cases developing more profound deficits. Compared with the previous cases, our patient preserved relatively much of her cognitive skills, further approaching the EPMR phenotype. Neurophysiological alterations in *CLN8*-associated diseases include EEG anomalies. A progressive background slowing is consistently reported. Interictal discharges include a spike-slow wave or polyspike-slow wave complexes and polyspikes [29,30,31,32,33,34]. Notably, epileptiform activity is scant in EPMR. Our patient’s recordings were dominated by background slowing, and she only recently developed unequivocal epileptiform transients. In imaging studies, cerebellar atrophy is the rule and involves both cerebellar vermis and hemispheres in the majority of cases [20,24,28,29,30,31,32,33,34,35,36]. Our patient is no different. Supratentorial, early cerebral atrophy is noted in most vLINCL cases, while it is a late feature in EPMR. Most of the patients bear white matter hyperintensities. At the age of 13, our patient shows no gross cerebral atrophy, although multiple white matter hyperintensities are present. On ultrastructural examination, there is much heterogeneity in both vLINCL and EPMR. The storage material may take the form of curvilinear and fingerprint profiles or granular osmophilic deposits, often intermixed in one patient [20,29,30,31]. Our patient’s presentation is similar to the EPMR phenotype, with epilepsy comprising the dominant symptom, relatively indolent progression, focal seizures, and lack of spontaneous myoclonus. On the other hand, it resembles the *CLN8*-related vLINCL described above, with developmental delay present from infancy and visual deterioration. In fact, this patient, together with other borderline cases, suggests a continuous spectrum of *CLN8*-associated phenotypes rather than a sharp distinction between EPMR and vLINCL. Figure 3 shows the proposed spectrum of NCL8 clinical phenotypes. The vast majority of mutations in all clinical phenotypes spectrum affect the large TLC domain of the protein.

*CLN8* encodes a ubiquitously expressed transmembrane protein of unknown function, mostly confined to the endoplasmic reticulum (ER) and ER-Golgi intermediate compartment [38]. Based on sequence homology, the CLN8 protein was classified as a member of the lipid-sensing protein family TLC containing human TRAM and yeast Lag1p. Thus, it may be involved in lipid synthesis and transport and act as a proteolysis regulator [39]. Eventually, the postulated pathogenic mechanisms lead to the formation of autofluorescent deposits containing subunit c of mitochondrial ATP synthase and sphingolipid activator proteins and to neuronal loss, not unlike the other NCLs [2,25]. While the autofluorescent deposits are indeed present in many tissue and cell types, they predominate in neural cells, and most cellular loss occurs there. This may partially be explained by the magnitude of *CLN8* expression in various tissue types. Furthermore, the protein’s function may be different in neurons and other cells. In non-neuronal cells, the protein localizes to the endoplasmic reticulum (ER) and ER-Golgi intermediate compartment, while in neurons, an extra-ER location was suggested [40]. Although NCLs show clinical variations in the disease progression, they all are still fatal. Therefore, enzyme replacement therapies are tried in the NCL group as in all lysosomal storage diseases. In 2017, cerliponase alpha gained FDA approval as the first enzyme replacement therapy to stop the progression of NCL2 [26].

## 5. Conclusions

We report a novel *CLN8* mutation as a cause for NCL8 in a girl with developmental delay and epilepsy, cerebellar syndrome, visual loss, and progressive cognitive and motor regression. This case further enriches the varied phenotype spectrum of pathogenic *CLN8* variants.

## Figures and Tables

**Table 1:** Classic NCL types [4]. Of note, the very first researcher to describe the disease in 1826, Stengel [5], remains unrecognized. AR, autosomal recessive; AD, autosomal dominant.

Type	Eponym	Original Reports	Age of Onset	Distinctive Clinical Features
Infantile	Haltia-Santavuori	Haltia and Santavuori [6]	0–2 y	Rapid psychomotor deterioration, seizures, visual impairment, purposeless hand movements (Rett-like). Death around age 10 y [1].
Late-infantile	Jansky-Bielschowsky	Jansky [7] and Bielschowsky [8]	2–4 y	Initially epilepsy, followed by cognitive deterioration, myoclonus, ataxia, vision impairment. Death by age 6–15 y.
Juvenile	Batten, Batten-Spielmeyer-Vogt	Stengel [5], Batten [9], Spielmeyer [10] and Vogt [11]	4–10 y	Initially rapidly progressive visual loss, followed by epilepsy. Death by age 30 y.
Adult	Kufs (AR), Parry (AD)	Kufs [12], Boehme [13]	15–50 y	Cognitive decline, ataxia, myoclonus, epilepsy, behavioral changes, no visual impairment

**Table 2:** Summary of clinical phenotypes of the described cases. GTCS—general tonic-clonic seizure, WM—white matter. ^‡^—excluding developmental delay.

Year/Phenotype	1994/EPMR	2000/EPMR	2017/EPMR	2001/vLINCL	2006/vLINCL	2010/vLINCL	2010/vLINCL	2010/vLINCL
**Authors**	Hirvasniemi et al. [20]	Ranta et al. [21]	Sahin et al. [24]	Mitchell et al. [22]	Cannelli et al. [28]	Reinhardt et al. [29]	Reinhardt et al. [29]	Reinhardt et al. [29]
**Nationality**	Finnish	Turkish	Turkish	Turkish	Italian	German	Pakistani	Turkish
**No of cases, sex**	23, M(11), F(12)	6	5, M(5)	5	3, M(3)	1, F(1)	1, F(1)	2, M(1), F(1)
**Mutation**	Homoz. c.70C>G, p.(Arg24Gly)	(1) Homoz. c.789G>C, p.(Trp263Cys); (2) Homoz. c.610C>T, p.(Arg204Cys); (3) Comp. heteroz. c.789G>C, p.(Trp263Cys)+c.610C>T, p.(Arg204Cys)	Homoz. c.677T>C, p. (Leu226Pro)	(1) Homoz. c.610C>T, p.(Arg204Cys); (2) Comp. heteroz. c.46C>A, p.(Leu16Met)+ c.509C>T, p.(Thr170Met); (3) Homoz. c.88delG, p.(Ala30Leufs20)	(1) Comp. heteroz c.581A>G, p.(Gln194Arg) + c.66delG, p.(Ala30Leufs*20); (2) Comp. heteroz c.473A>G, p.(Tyr158Cys) +c.66delG, p.(Ile23Serfs*5); (3) Homoz. c.88G>C, p.(Ala30Pro)	Homoz. c.611G>T, p.(Arg204Leu)	Homoz. c.709G>A, p.(Gly237Arg)	Homoz. c.544-2566_590del2613, p.0
**Infancy psychomotor development**	Normal (20/23), Delayed (3/23)	N/A	Normal (5/5)	Delayed	Delayed	Normal	N/A	Delayed
**Age of symptoms onset** ^‡^	6.7 y (5–10)	5.7 (3,5–7)	8.4 y (8–10)	3.5 (3–4)	4.5 y (3,5–6)	3.5 y	4 y	3.25 y (3–3,5)
**First symptoms** ^‡^	Seizures	N/A	Seizures	Mixed	Seizures	Attention, sleep, speech disturbance	Ataxic gait	Seizures
**Epilepsy**	Yes, GTCS (23/23), complex focal (7/23)	Yes (6/6)	Yes, GTCS (5/5)	Yes (4/4)	Yes, myoclonic (2/3), tonic-clonic (1/3)	Yes, absence	Yes, drop attacks	Yes, myoclonic and drop attacks
**Epilepsy onset**	6.7 y (5–10)	6.2 y (3.5–8)	8.4 y (8–10)	3.5 y (3–4)	4.5 y (3.5–6)	4 y	6,5 y	3.25 y (3–3.5)
**Cognitive deterioration and onset**	Yes (23/23), 2–5 y, after epilepsy onset	Yes (6/6)	Yes (5/5), 6.5 y (5–7y), after epilepsy onset	Yes	Yes	Yes, 3.5y	Yes, After ataxia onset	Yes, After epilepsy onset
**Cognitive outcome**	Borderline (4/19), Mild (4/19), Moderate (5/19), Severe (6/19)	N/A	Mild (3), Moderate (1), Severe (1)	N/A	N/A	Severe	Severe	Severe
**Ataxia and onset**	Yes (16/19), <30 y	yes (5/5), 8.2 y (4–12)	yes (5/5)	yes, 3.8 y (3.5–4)	yes	yes, 4 y	yes, 4 y	yes, 4 y
**Abnormal speech onset**	N/A	8.6 y (7–9)	16 y (15–18)	N/A	N/A	4 y	5.5 y	4 y
**Abnormal gait onset**	<30 y	N/A	14.6 y (14–16)	N/A	N/A	N/A	N/A	N/A
**Myoclonus and onset**	No	Yes (4/4), 7.1 y (3.5–9)	No	Yes, 3.9 y (3–4.5)	Yes	Yes, 5 y	Yes, 5 y	Yes, 4 y
**Visual loss and onset**	No or minor	Yes (6/6), 7.5 y (5–9)	No	Yes, 3.6 y (3–4)	Yes	Yes, 9 y	Yes, 7 y	Yes, 6 y
**Optic pathology**	None	N/A	None	N/A	N/A	Retinal degeneration	Optic nerve atrophy	Retinal degeneration
**Progression rate**	Slow	Rapid	Slow	Rapid	Rapid	Rapid	Rapid	Rapid
**EEG**	Slow background, disappearance of sleep patterns, scanty interictal epileptiform activity	N/A	Normal	N/A	N/A	N/A	N/A	Generalized polyspike slow wave
**Cerebral MRI**	Atrophy at later stages	N/A	Atrophy	N/A	Atrophy	Cortical atrophy	Atrophy, T2/FLAIR PV WM hyperintensivities	Atrophy, T2/FLAIR WM hyperintensivities
**Cerebellar atrophy**	Yes	N/A	Yes	N/A	Yes	Yes	Yes	Yes
**Other**	Behavioural difficulties in puberty (11/23)	N/A	N/A	N/A	Patients with deletions: earlier onset, myoclonus more prominent	N/A	Noise elicited myoclonus	N/A
**Year/Phenotype**	**2012/vLINCL**	**2012/vLINCL**	**2016/vLINCL**	**2018/vLINCL**	**2020/vLINCL**	**2015/cNCL**	**2016/aLINCL**	**2021/aLINCL**
**Authors**	Mahajnah et al. [30]	Allen et al. [31]	Katata et al. [32]	Gao et al. [33]	Alkhars et al. [34]	Kohan et al. [35]	Sanchez et al. [36]	Badura-Stronka et al.
**Nationality**	Israeli/Arabian	Irish	Japanese	Chinese	Arabian	Argentinian	Hispanic	Polish
**No of cases, sex**	3, M(2), F(1)	1, M(1)	1, M(1)	1, M(1)	2, M(1), F(1)	1, F(1)	2, M(1), F(1)	1, F(1)
**Mutation**	Homoz. c.763C>G, p.(Gln255*)	Comp. heteroz. c.562_563delCT, p.(Leu188Valfs*58) + 8p23.3 terminal deletion	Homoz. c.620T>G, p.(Leu207Arg)	Comp. heteroz. c.298C>T, p.(Gln100*) + c.551G>A, p.(Trp184*)	(1) Homoz. c.699_700delGT, p.Phe234Profs12; (2) Homoz. c.(?_-1)_(5431_544-1)del	(rs143730802) + (rs587779411)	Comp. heteroz. c.200C>T, p.(Ala67Val) + *CLN8* deletion	Homoz. c.531C>T, p.(Trp177Cys)
**Infancy psychomotor development**	Normal	Delayed	Delayed	Normal	Normal (1/2), Delayed (1/2)	Delayed	Normal	Delayed
**Age of symptoms onset** ^‡^	5 y (4–6)	4 y	3 y	4 y	2 y	Congenital	11.5 (7–16)	5.5 y
**First symptoms** ^‡^	Seizures (2/3), Mixed (1/3)	Ataxia	Seizures	Seizures	Seizures (1/2), Ataxia (1/2)	Seizures	Seizures (1/2), Visual (1/2)	Seizures
**Epilepsy**	Yes, tonic-clonic	Yes	Yes, drop attacks, myoclonic	Yes	Yes, GTCS, myoclonic	Yes, GTCS	Yes, GTCS	Yes, GTCS, complex focal
**Epilepsy onset**	6.7 y (5–9)	4.5 y	3 y	4 y	1.5 y (1–2)	3 y	11.5 y (7–16)	5.5 y
**Cognitive deterioration and onset**	Yes, After epilepsy onset (2/3)	Yes, 4 y	Yes, N/A	Yes, 3 y, after epilepsy onset	Yes, 3 y, after epilepsy onset (1/2), Birth (1/2)	Yes, Birth	No, N/A	Yes, <2 y, after epilepsy onset
**Cognitive outcome**	Profound (1/3), Moderate (1/3), Mild (1/3)	N/A	N/A	N/A	Severe	Severe	Excellent	Moderate
**Ataxia and onset**	yes (1/3), 5 y, No (2/3)	yes, 4 y	yes, 4 y	yes, 7 y	yes, 2.5 y (1–4)	No	yes	yes, 6.6 y
**Abnormal speech onset**	N/A	4 y	N/A	7 y	3 y (1/2)	N/A	N/A	3 y
**Abnormal gait onset**	N/A	4 y	4 y	7 y	3 y (2-4)	Never achieved	N/A	9.5 y
**Myoclonus and onset**	Yes (1/3)	N/A	Yes, 5 y	N/A	Yes (2/2), 1.5 y (1–2)	Yes, 6 y	No	No
**Visual loss and onset**	Yes, 6.5 y (6–7.5)	Yes, 5.5 y	Yes, 5 y	Yes, 7 y	Yes (2/2), 2.5 y (2–3)	N/A	Yes, 9 y (5–13)	Yes, 9.5 y
**Optic pathology**	Retinal degeneration, Optic nerve atrophy (2/3)	Abnormal ERG	Retinal and macular	N/A	Optic nerve atrophy	N/A	Complex macular (1/2), Retinal and optic nerve atrophy (1/2)	Macular dystrophy, optic nerve atrophy
**Progression rate**	Rapid (1/3), Slow (2/3)	Rapid	Rapid	Rapid	Rapid	Rapid	Slow	Slow
**EEG**	Slow background (1/3), Multiple spikes and bursts of generalized spike-slow wave (1/3)	Slow background, complex focal seizures	Diffuse spikes and slow waves	Slow background, abundant generalized atypical spike-slow wave	Generalized slow background with polyspike epileptiform discharges	N/A	N/A	Progressive slowing, paroxysmal slow waves; generalized sharp-slow wave after 12 y
**Cerebral MRI**	Diffuse atrophy (1/3), Mild atrophy (1/3), T2/FLAIR WM hyperintensivities (2/3)	T2/FLAIR posterior deep WM hyperintensivities	T2/FLAIR diffuse PV WM hyperintensivities	Diffuse atrophy	Diffuse atrophy (1/2)	N/A	N/A	T2/FLAIR WM hyperintensivities
**Cerebellar atrophy**	Yes	Yes	Yes	Yes	Yes	Yes	N/A	Yes
**Other**	Severily affected, rapid deterioration (1/3)	N/A	N/A	N/A	N/A	N/A	Polidactyly, ADHD (1/2)	Myoclonus on lamotrigine; hypersensitive to noise

## Data Availability

The data presented in this study are available on request from the corresponding author.
